# Paraneoplastic Cerebellar Syndrome Revealing Human Epidermal Growth Factor Receptor 2 (HER2)-Positive Breast Cancer: A Case Report

**DOI:** 10.7759/cureus.98519

**Published:** 2025-12-05

**Authors:** Sara Bendadi, Leila Afani, Othmane Zouiten, Mohamed El Fadli, Rhizlane Belbaraka

**Affiliations:** 1 Medical Oncology, Mohammed VI University Hospital, Marrakesh, MAR; 2 Oncology, Mohammed VI University Hospital, Marrakesh, MAR

**Keywords:** anti-yo antibody, her2-positive breast cancer, immune checkpoint inhibitor, immuno-clinical profiles, paraneoplastic cerebellar degeneration

## Abstract

Paraneoplastic cerebellar degeneration (PCD) is a rare neurological disorder that can precede the diagnosis of an underlying malignancy by several months or even years. We report the case of a 46-year-old woman who presented with progressive gait instability, dysarthria, and nystagmus. Brain magnetic resonance imaging (MRI) revealed cerebellar atrophy without any mass lesion, and serologic testing showed strong anti-Yo antibody positivity. Two years later, she was diagnosed with a human epidermal growth factor receptor 2 (HER2)-positive, hormone receptor-negative invasive ductal carcinoma of the breast. The patient underwent a modified radical mastectomy followed by adjuvant chemotherapy and trastuzumab. Despite appropriate oncologic treatment, her neurological symptoms persisted and gradually worsened. This case highlights the diagnostic challenge of PCD, particularly when neurological manifestations precede tumor detection, and suggests that HER2-positive breast cancer may also be associated with anti-Yo antibodies. Early recognition of this syndrome and systematic malignancy screening are essential to improve patient outcomes.

## Introduction

Paraneoplastic neurological syndromes (PNS) are a heterogeneous group of rare disorders that, by definition, are associated with malignancies but are not caused by direct tumor invasion, metastasis, metabolic or nutritional deficits, infections, or drug toxicity [1]. PNS may occur concomitantly with or after a cancer diagnosis; however, in many cases, they represent the first manifestation of an underlying malignancy, sometimes appearing months or even years before tumor detection [1].

Several clinical forms have been described, including paraneoplastic cerebellar degeneration (PCD), limbic encephalitis, encephalomyelitis, subacute sensory neuropathy, Lambert-Eaton myasthenic syndrome, opsoclonus-myoclonus syndrome, dermato- or polymyositis, pandysautonomia, and cancer-associated retinopathy [2].

Among these, PCD is one of the most frequent, typically associated with gynecological and breast malignancies [3]. We report the first documented case of anti-Yo antibody-positive paraneoplastic cerebellar syndrome revealing breast cancer at the Onco-Hematology Department of the University Hospital of Marrakech.

## Case presentation

A 46-year-old woman, divorced and mother of two, presented with a three-month history of progressive gait instability and limb incoordination. She also reported mild dysarthria for four months and intermittent nystagmus for two months. Neurological examination revealed marked cerebellar ataxia requiring assistance for ambulation, bilateral abducens nerve involvement, and pyramidal signs, while muscle strength and sensation were preserved.

Initial investigations, including brain magnetic resonance imaging (MRI), infectious and nutritional laboratory testing, and cerebrospinal fluid analysis, were unremarkable. In December 2021, a paraneoplastic antibody panel demonstrated strong anti-Yo antibody positivity, supporting the diagnosis of PCD. No underlying malignancy was identified despite a first systemic evaluation, and the patient was subsequently lost to follow-up.

Nearly two years later, she re-presented with worsening neurological symptoms. A left breast mass was detected on clinical examination, and subsequent imaging and biopsy confirmed human epidermal growth factor receptor 2 (HER2)-positive, hormone receptor-negative invasive ductal carcinoma. She underwent a modified radical mastectomy with axillary lymph node dissection. Postoperatively, a repeat brain MRI showed cerebellar atrophy consistent with advanced PCD. The patient was then started on adjuvant chemotherapy with taxanes and trastuzumab (Table 1).

**Table 1:** Immunoallergology test for autoimmune antibodies associated with the nervous system. The bold texts indicate abnormal or positive results for each antibody tested. Anti-Yo: anti-Purkinje cell cytoplasmic antibody type 1; Ac anti-CV2: anti-collapsin response mediator protein-5 (CRMP-5) antibody; Ac anti-PNMA2 (Ma2/Ta): anti-PNMA2/Ma2/Ta neuronal antibody; Ac anti-Ri: anti-neuronal nuclear antibody type 2; Ac anti-Hu: anti-neuronal nuclear antibody type 1; Ac anti-SOX1: anti-SRY (sex-determining region Y)-box 1 antibody; Ac anti-Zic4: anti-Zic family member 4 antibody; Ac anti-GAD65: anti-glutamic acid decarboxylase 65 antibody; Ac anti-Tr (DNER): anti-Tr (Delta/Notch-like EGF-related receptor) antibody

Searched antibodies	Results
Ac anti-amphiphysin	Negative
Ac anti-CV2	Negative
Ac anti-PNMA2 (Ma2/Ta)	Negative
Ac anti-Ri	Negative
Ac anti-Yo	Highly positive
Ac anti-Hu	Negative
Ac anti-recoverin	Negative
Ac anti-SOX1	Negative
Ac anti-titin	Negative
Ac anti-Zic4	Negative
Ac anti-GAD65	Negative
Ac anti-Tr (DNER)	Negative

A comprehensive clinical and imaging assessment, including gynecological, abdominal, and thoracic evaluation, was unremarkable. Whole-body computed tomography (CT) did not reveal any malignancy. Positron emission tomography (PET)-CT was indicated but could not be performed due to socioeconomic limitations and unavailability at the University Hospital of Marrakech. As no tumor was identified at that time, the patient was subsequently lost to follow-up.

Nearly two years later, she re-presented with worsening neurological symptoms and a decline in general condition. Examination showed an Eastern Cooperative Oncology Group (ECOG) performance status of 2 with persistent pyramidal signs, progression of the cerebellar syndrome, and worsening dysarthria. Breast examination revealed a firm, mobile, painless 3 × 3 cm mass in the left inframammary sulcus. Breast ultrasound and mammography were classified as Breast Imaging Reporting and Data System (BIRADS) 4. A Tru-Cut biopsy confirmed invasive ductal carcinoma, Scarff-Bloom-Richardson (SBR) II, with vascular emboli. Immunohistochemistry demonstrated HER2 overexpression, estrogen receptor negativity (ER 0%), progesterone receptor negativity (PR 0%), and a Ki-67 index of 50%.

**Figure 1:**
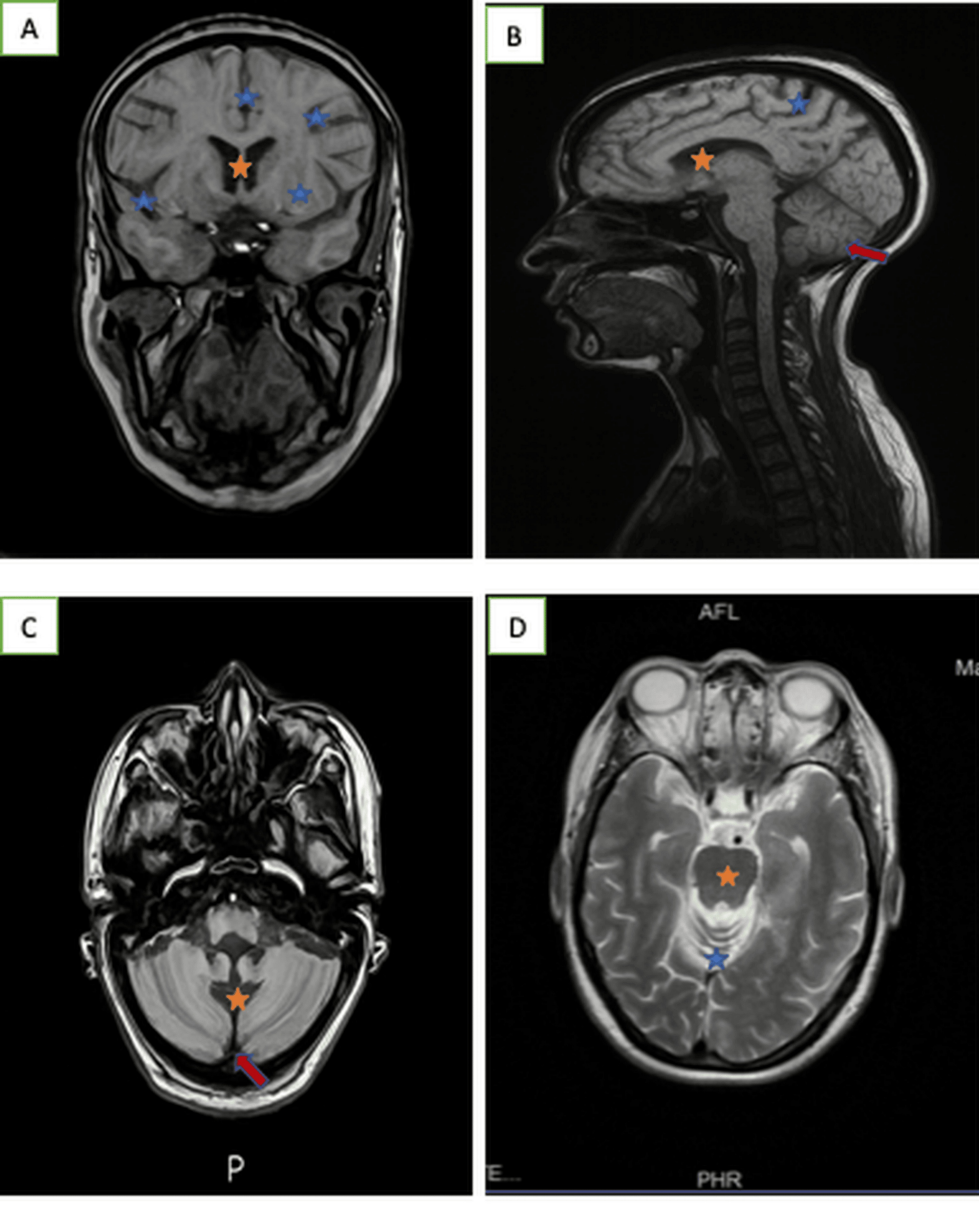
Brain MRI in different planes ((A) coronal cerebral T1-weighted sequence, without contrast, (B) median sagittal T1-weighted sequence, (C) axial T1-weighted sequence, (D) axial T2-weighted sequence) showing enlargement of the cerebellar sulci (blue stars) and folia with dilatation of the fourth ventricle (orange stars), consistent with diffuse cerebellar atrophy (red arrows) in the context of paraneoplastic cerebellar degeneration. MRI: magnetic resonance imaging; AFL: anterior funicular lesion; PHR: posterior horn region

The patient underwent a modified radical mastectomy (Patey procedure) with axillary lymph node dissection in October 2021. Staging showed no distant metastases (pT2N0M0). In January 2022, a repeat brain MRI revealed cortico-subcortical and cerebellar atrophy with enlargement of the subarachnoid spaces, fourth ventricle, and basal cisterns, findings consistent with advanced PCD. A multidisciplinary tumor board recommended adjuvant chemotherapy with taxanes combined with trastuzumab (Figure 1).

## Discussion

PCD is one of the most disabling neurological paraneoplastic syndromes, characterized by subacute onset and progressive deterioration. It is most frequently linked to breast and gynecological cancers and commonly associated with anti-Yo antibodies, as in our patient.

In this case, the nearly two-year interval between neurological onset and tumor diagnosis highlights the diagnostic challenge of PCD. Although anti-Yo antibody positivity provided an early paraneoplastic clue, the initial absence of radiologic findings and the lack of identifiable malignancy delayed the diagnosis. Similar cases have been described in which PCD preceded tumor detection by several months or even years [4,5].

Interestingly, our patient had a HER2-positive, hormone receptor-negative breast carcinoma. While anti-Yo-associated PCD typically occurs in hormone receptor-positive cancers, emerging evidence suggests that HER2-positive subtypes may also trigger paraneoplastic immune responses, though less commonly [3]. This raises questions about the differential immunogenicity of breast cancer phenotypes.

Despite adequate oncologic treatment, the neurological course remained severe and progressive. This aligns with prior observations indicating that neurological recovery is uncommon, even when the underlying tumor is successfully managed [6,7]. The poor prognosis likely reflects irreversible immune-mediated Purkinje cell destruction within the cerebellum.

This case underscores the need to consider a paraneoplastic etiology in patients, particularly middle-aged women, presenting with subacute cerebellar syndromes. Early antibody screening (notably anti-Yo) can guide investigations toward an occult neoplasm even when imaging is initially negative. Comprehensive systemic evaluation and prolonged follow-up are essential in such scenarios.

Overall prognosis remains poor: in one large series, only 21% of anti-Yo-positive patients regained independent ambulation, with most remaining severely disabled despite cancer remission [8]. Outcomes in HER2-positive cases treated early appear slightly better but remain unsatisfactory. To our knowledge, this represents the first documented case in Morocco.

## Conclusions

This report highlights the importance of considering PCD in patients presenting with subacute cerebellar symptoms. Detection of anti-Yo antibodies should prompt thorough malignancy screening and repeat evaluations when initial imaging is inconclusive. Early cancer management, particularly in HER2-positive breast cancer, may slow neurological decline, though functional recovery remains rare once Purkinje cell loss has occurred. Wider access to PET-CT imaging and earlier initiation of immunotherapy could improve outcomes and quality of life, even when neurological deficits persist. Case aggregation and further studies are required to refine prognostic indicators and optimize multidisciplinary management.
